# Quality of life changes with canakinumab therapy in adults with colchicine resistant FMF

**DOI:** 10.1186/1546-0096-13-S1-P89

**Published:** 2015-09-28

**Authors:** A Gul, H Özdogan, O Kasapcopur, B Erer, S Ugurlu, S Sevgi, S Turgay

**Affiliations:** 1 İstanbul University Faculty of Medicine, İstanbul, Turkey; 2 Cerrahpasa Faculty of Medicine, İstanbul, Turkey; 3 Novartis Pharma, Turkey, Turkey

## Introduction

Familial Mediterranean Fever (FMF), the most common form of the hereditary autoinflammatory disorders, is characterized by recurrent attacks of fever along with serosal or synovial inflammation lasting usually 12 to 72 hours. FMF is associated with impaired functional ability, and the persistent disabling features and chronic pain, emotional and physical limitations can have a negative impact on the health-related quality of life (QoL) of the patients.

There is no established treatment available for those resistant or intolerant to standard of care colchicine treatment. Interleukin-1 (IL-1) plays a pivotal role in the pathogenesis of crFMF. Canakinumab, a fully human, selective, anti-IL-1β monoclonal antibody, binds to IL-1β and inactivates its signalling activity. Gul et al. have described the efficacy and safety of canakinumab in adults with colchicine resistant (cr) FMF in a local pivotal phase II trial. Here, we report the effect of canakinumab treatment on QoL measured by SF-36 Questionnaire.

## Objectives

This study aimed to show the effects of canakinumab treatment on quality of life by 8 sub-items of SF-36 as well as to see the correlation between the Physician Global Assessments (PGA) and SF-36 scores.

## Methods

9 crFMF patients with ≥1 attack/month in the preceding 3-months despite the highest tolerated colchicine dose entered the study. Canakinumab injections were administered at Day 1, Day 29 and Day 57. Changes in the quality of life was recorded in 9 subjects by using SF-36 at Day 1, 8, 29, 57, 86, 115 and the end of the study.

## Results

In all 8 items (physical functioning, role limitations due to emotional/physical health, energy, emotional-well being, body pain, social functioning and general health) of SF-36 scores improved dramatically with canakinumab treatment, starting from 1st day. However the differences in emotional well-being and role limitations due to emotional problems scores couldn't reach the statistical significance. All scores are showed in tables below. Also there was a strong negative correlation between Physician Global Assessment (PGA) and Physical Component Score (R-sq: -0,793). A weaker correlation observed between Mental Component Score and Physical Global Assessment (R-sq: -0,540).

## Conclusion

Canakinumab treatment in cr-FMF patients resulted in a rapid improvement in QoL measures. Also these improvements sustained during the withdrawal period. PGA scores appear to be in compliance with the Physical and Mental Component scores of the SF-36 questionnaires. A stronger correlation was observed for the Physical Component Score.

